# Clinical outcomes and surgical applications of 3D-printed patient-specific implants for traumatic bone defects: a scoping review

**DOI:** 10.1186/s13018-026-07064-z

**Published:** 2026-07-29

**Authors:** David Slawaska-Eng, Nicholas Garbuz, Matthew Mellon, James Yan, Emilie Sandman, Derek H. Rosenzweig, Paul A. Martineau

**Affiliations:** 1 Department of Orthopaedic Surgery, Keck School of Medicine, University of Southern California, 1520 San Pablo St, Suite #2000, Los Angeles, CA 90033 USA; 2 Michael G. DeGroote School of Medicine, McMaster University, Hamilton, ON Canada; 3 Department of Surgery, Division of Orthopaedic Surgery, McMaster University, Hamilton, ON Canada; 4 Department of Surgery, Division of Orthopaedic Surgery, Université de Montréal, Montréal, QC Canada; 5 Department of Surgery, Division of Orthopaedic Surgery, McGill University, Montréal, QC Canada

**Keywords:** Three-dimensional printing, Additive manufacturing, Patient-specific implant, Traumatic bone defect, Orthopaedic trauma, Bone reconstruction

## Abstract

**Purpose:**

Traumatic bone defects are complex injuries associated with prolonged disability and limited reconstructive options. Three-dimensional printed (3DP) patient-specific implants provide anatomically matched, mechanically tailored solutions. This review synthesizes current clinical evidence regarding their use, effectiveness, and safety in trauma-related bone defects.

**Methods:**

MEDLINE, Embase, and Scopus were searched from inception to February 17, 2025. Human studies reporting clinical outcomes of custom 3DP implants for traumatic bone defects were included. Two reviewers independently screened studies, extracted data, and assessed methodological quality using the MINORS tool. Owing to heterogeneity in anatomy, defect characteristics, adjunctive procedures, and outcome definitions, a meta-analysis was not performed.

**Results:**

Twelve studies comprising 108 patients met inclusion criteria; most were retrospective, non-comparative case series (predominantly Level IV evidence). Titanium-based implants were most common (92 patients), followed by tantalum (9) and polyetheretherketone (5). Defect sites included the humerus, forearm, femur, tibia, and calcaneus. Union assessment methods varied, but confirmed union was frequently reported. For tibial defects, union was confirmed in 13/20 patients; four had indeterminate imaging and three declined a planned second-stage procedure. Functional outcomes generally improved. Reported complications were typically manageable, and no catastrophic implant failures were described, although follow-up duration was limited.

**Conclusion:**

Current evidence suggests that patient-specific 3DP implants are a feasible reconstructive option for selected traumatic bone defects. However, evidence certainty remains very low, and prospective comparative studies with standardized reporting are required.

**Level of evidence:**

IV (systematic review of primarily Level IV case series and retrospective studies).

**Supplementary Information:**

The online version contains supplementary material available at https://doi.org/10.1186/s13018-026-07064-z.

## Introduction

Bone defects represent osseous injuries that can often exceed the body’s intrinsic capacity for spontaneous healing and typically require surgical reconstruction for successful union [1]. In the setting of trauma, these defects commonly arise from high-energy injuries, open fractures, severe comminution, or extensive debridement following infection [1]. Trauma-related bone loss, particularly when segmental. is associated with high nonunion rates and morbidity, often requiring advanced reconstruction [1].

Traditional treatment modalities, including autografts, allografts, and bone graft substitutes, remain widely utilized but are frequently limited by donor site morbidity, graft availability, and variable incorporation [2–4]. Autologous bone grafting alone is generally reserved for smaller defects, while larger or biologically compromised defects often require staged reconstruction or adjunctive techniques [5]. The Masquelet induced membrane technique has demonstrated success in selected cases; however, outcomes remain variable, particularly in extensive defects or in the presence of infection [6, 7]. Other reconstructive strategies include distraction osteogenesis, which encompasses bone transport techniques as well as acute limb shortening followed by subsequent lengthening, in addition to vascularized bone grafts using microsurgical techniques and structural reconstruction with bone grafting supported by devices such as mesh cages [8]. These approaches underscore the complexity of managing traumatic bone loss and highlight the need for alternative reconstructive solutions.

Three‑dimensional printing, also known as additive manufacturing, fabricates objects layer by layer from digital models [9]. Originally developed for industrial prototyping in the 1980s, this technology has expanded into medical device production [9, 10]. Recent studies have demonstrated the feasibility of three-dimensional printed (3DP) orthopedic implants in the management of bone loss and infection models [10–13]. Clinically translated implants have been manufactured using electron beam melting (EBM) and selective laser melting (SLM) systems and sterilized via standard steam autoclaving or gamma irradiation [14, 15]. These devices are produced from biocompatible metals such as titanium (Ti) alloys (Ti‑6Al‑4 V) and tantalum (Ta), allowing the fabrication of porous structures that encourage bone ingrowth and incorporation [10, 14, 15].

Advancements in three-dimensional printing have introduced novel reconstructive approaches in orthopaedic trauma surgery, offering patient-specific solutions for complex bone defects [6, 16, 17]. This technology enables the fabrication of anatomically precise implants, scaffolds, and surgical guides tailored to the unique structural and biomechanical characteristics of each patient [7, 18]. Preoperative planning facilitated by 3D printing has been shown to improve surgical accuracy, reduce intraoperative time, and enhance overall patient outcomes [19, 20]. Nevertheless, the long‑term viability, cost‑effectiveness, and regulatory considerations of 3DP orthopedic implants remain areas of active investigation [18, 21] and pose significant challenges in translating 3D printing from experimental preclinical studies into routine clinical use for trauma-related bone defects.

The objective of this review is to synthesize the available clinical evidence regarding the adoption, effectiveness, safety, and patient-reported outcomes of custom three-dimensional printed implants for reconstruction of traumatic bone defects. In addition, we identified limitations within the current literature and areas requiring further investigation to guide future clinical application.

## Methods

This systematic review was conducted in accordance with the Preferred Reporting Items for Systematic Reviews and Meta-Analyses guidelines [22]. The protocol was prospectively registered on PROSPERO (CRD420251056502) [23]. A completed PRISMA-ScR checklist is provided as supplementary material in Appendix 1 (Table A4).

### Search strategy

MEDLINE, Embase, and Scopus were searched from inception to February 17, 2025. The strategy combined MeSH terms and free-text keywords related to 3DP implants, additive manufacturing, and bone defects. Full search strategies are provided in Appendix 1 (Tables A1-A3). Trauma etiology was confirmed during full-text screening, and only studies involving acute traumatic bone loss, post-traumatic infection, or traumatic nonunion were included.

### Eligibility criteria

Studies were included if they met the following predefined inclusion criteria: (1) human studies; (2) clinical application of custom 3DP implants for the treatment of trauma-related bone defects, including post-traumatic infection or traumatic nonunion; and (3) reporting of quantitative or qualitative clinical outcomes. Studies were excluded if they met any of the following criteria: (1) non-human or in vitro studies; (2) non-English or non-French publications; (3) review articles; (4) case reports; and (5) studies lacking explicit clinical outcome data.

### Study selection

Two reviewers (N.G. and M.M.) independently screened titles, abstracts, and full texts. Disagreements were resolved by discussion with third-reviewer (D.S.E.) adjudication when required. Inter-rater reliability was assessed using Cohen’s kappa statistic [24].

### Data extraction

A structured data extraction form was employed to systematically collect relevant study data using a shared document on Google Sheets (Google LLC, Mountainview, CA, USA). Extracted variables included study characteristics, patient demographics, defect characteristics, implant material, manufacturing method, union status, complications, and functional outcomes. Because definitions of union varied across studies, union was recorded according to each study’s reported criteria, typically based on radiographic or computed tomography evidence of bridging bone. When imaging findings were described as indeterminate or when patients declined planned staged procedures, these cases were recorded separately and not assumed to represent confirmed union. To ensure accuracy and consistency, the extracted data were cross-verified by a second reviewer.

### Quality assessment

The Methodological Index for Non-Randomized Studies (MINORS) was used to assess the methodological quality and risk of bias in all included studies [25]. MINORS assigns one of three scores to each criterion: 0 if not reported, 1 if poor quality, and 2 if high quality. Eight criteria are assessed for all included studies, including the appropriateness of the study aim, inclusion criteria, endpoints, follow-up, and prospective data collection and sample size calculation. Two reviewers (N.G, M.M.) independently performed the quality assessment. Agreement between reviewers was quantified using Cohen’s kappa statistic to measure inter-rater reliability. Discrepancies were subsequently resolved through structured discussions and adjudication.

### Data synthesis and statistical analysis

Given heterogeneity in study design, anatomy, defect metrics, adjunctive procedures, and outcome measures, a narrative synthesis was performed. Descriptive statistics were used to summarize reported outcomes where appropriate. A meta-analysis was not conducted due to variability and limited study quality. Findings were synthesized to identify patterns in reported outcomes and to highlight limitations in the current evidence base.

### Data availability and ethics statement

As this study synthesizes previously published data, institutional ethics review board approval was not required. All data underlying this review are derived from previously published studies and are available in the public domain.

The authors declare that no funds, grants, or other financial support were received during the preparation of this manuscript.

## Results

### Study selection

**Fig. 1:**
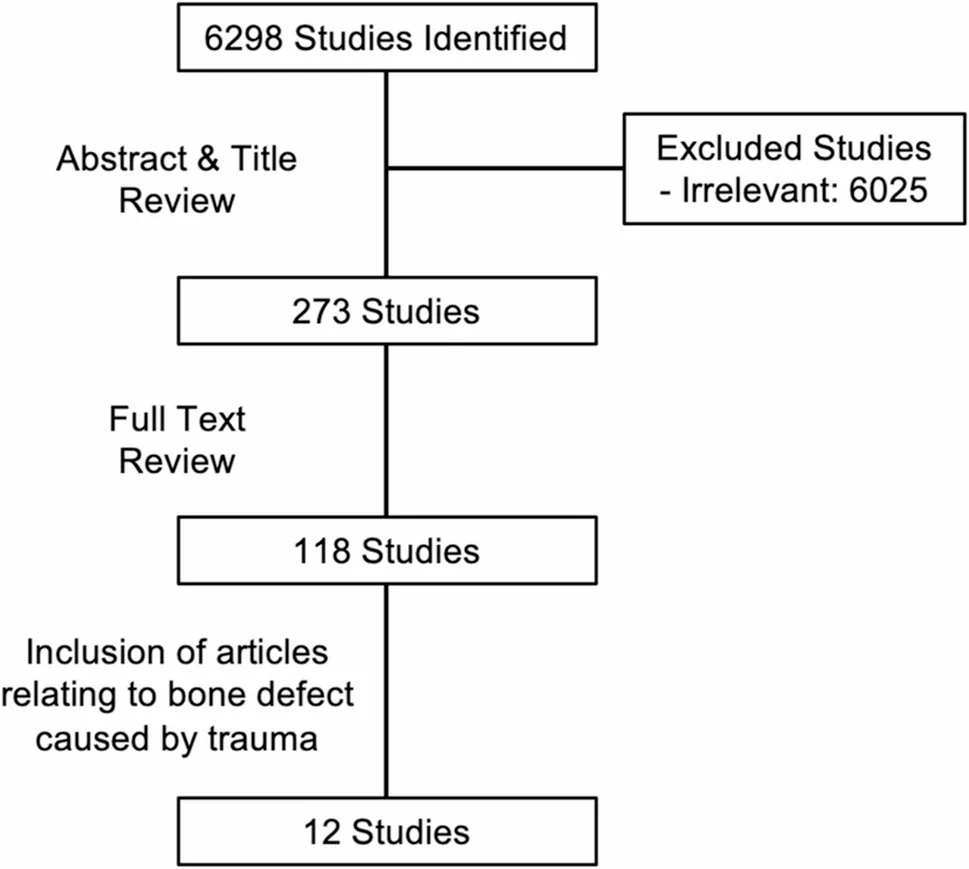
Flow diagram demonstrating the search strategy for custom 3D-printed implants for bone defect repair in defects caused by trauma

A total of 12 studies were included [26–37]. Agreement between the reviewers was substantial at the title and abstract (k = 0.72; 95% CI 0.68–0.76), as well as full text (k = 0.62; 95% CI 0.51–0.72) stages. The PRISMA flowchart is outlined in Fig. 1. A completed PRISMA-ScR checklist is provided as supplementary material.

### Study demographics

**Fig. 2:**
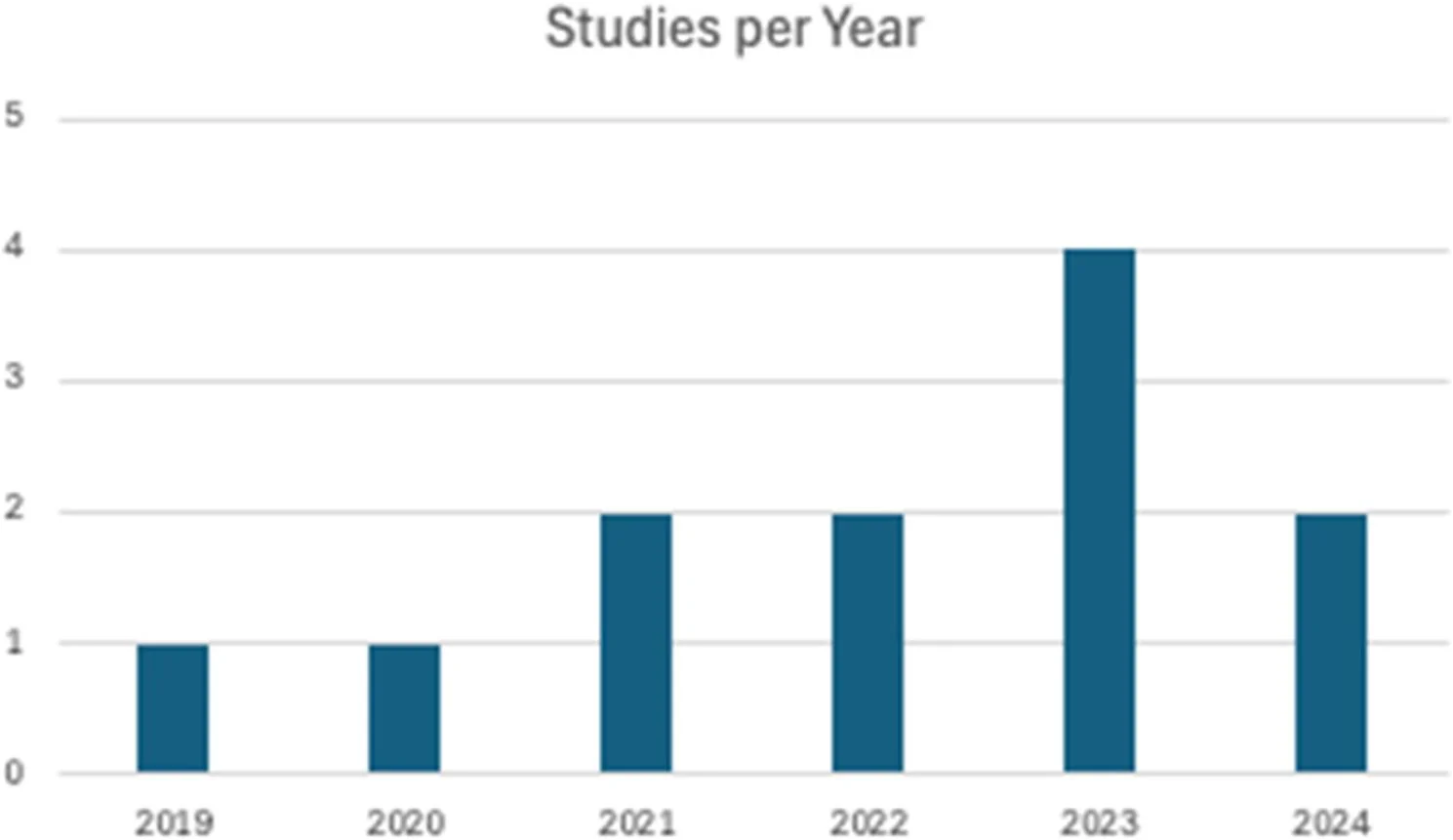
Studies included in this review versus year of publication

There is a positive trend in the number of publications over time as shown in Fig. 2. Geographic location of first authors appears to be centered primarily in China (7 studies), with studies published in South Africa (1), Ukraine (1), Turkey (1), Australia (1) and the United States (1). Eleven of the 12 included studies were retrospective, non‑comparative case series, corresponding to Level IV evidence.

### Study characteristics

There were 12 studies reporting the use of custom 3DP implants for traumatic bone defects (Table 1). Titanium-based implants were used in 10 studies, while polyetheretherketone and tantalum were each used in one study. Across all studies, 108 patients were included.

**Table 1:** Characteristics of included studies [26–37]

Author (Year)	Anatomical Location	Implant Material (Manufacturing Method, if reported)	Implant Type	Bone Graft	Number of Patients	Mean Bone Defect Size (L = cm, V=cm^3^)	Mean Follow Up (months)	Female (%)	Mean Age (years)
Gamieldien (2023) [33]	Femur (*n* = 7), Tibia (*n* = 2)	Titanium (EBM)	Scaffold (Masquelet)	Autograft	9	Femur: 12.4 (L) Tibia: 4.8 (L)	11.3	22	35
Hou (2020) [34]	Femur	Titanium (EBM)	Scaffold	None	5	12 (L)	16.4	60	52.6
Liu (2022) [32]	Femur (*n* = 5), Tibia (*n* = 6)	Titanium (SLM)	Scaffold	None	10	Femur: 12.6 (L) Tibia: 11.9 (L)	23.0	30	50.7
Liu (2024) [36]	Tibia	Titanium (SLM)	Scaffold	None	4	16.93 (L)	27.3	25	52.5
Lurin (2024) [30]	Humerus	PEEK	Scaffold	Allograft	5	> 10 (L)	14.8	0	33.8
Rao (2023) [27]	Distal radius (*n* = 1), Ulna midshaft (*n* = 1)	Metal*	Scaffold	Autograft	2	NR	NR	NR	47
Safali (2023) [35]	Radial head (*n* = 1), Distal humerus (*n* = 1), Acetabulum (*n* = 1) Mandibula (*n* = 1)	Titanium	Implant	None	4	NR	24.0	50	45.5
Tetsworth (2019) [29]	Femur	Titanium	Scaffold (Masquelet)	Auto + Allograft	5	14.0 (L), 192.4 (V)	21.8	40	49
Wang (2021) [28]	Humerus (*n* = 1), Tibia (*n* = 8), Femur (*n* = 7), Radius (*n* = 2), Clavicle (*n* = 1)	Titanium	Spacer (IM nail)	None	19	Humerus: 12.9 (L) Tibia: 6.7 (L) Femur: 9.2 (L) Radius: 1.1 (L) Clavicle: 2.5 (L)	22.7	21	44.7
Wu (2022) [31]	Femur	Tantalum	Scaffold	None	9	16.1 (L)	16.9	33	39.7
Zhang (2021) [37]	Femur	Titanium (EBM)	Scaffold	None	26	7.48 (L)	28.4	67	47.7
Zhao (2023) [26]	Calcaneus	Titanium	Scaffold (Masquelet cage)	Autograft	10	18.0 (V)	18.5	30	37

*NR* not reported, *PEEK* polyetheretherketone, *SLM* selective laser melting, *EBM* electron beam melting, *IM* intramedullary

* = Metal (specific alloy not reported)

### Implant classification and use of bone grafting

Based on detailed review of included studies, 3DP constructs were classified according to their reconstructive role: (1) osseointegrative scaffolds/implants used as structural cages, with or without adjunctive bone grafting; and (2) spacer-type constructs used primarily for temporary defect management (Table 1). Among the included studies, most implants functioned as structural scaffolds for intercalary defect reconstruction. Adjunctive bone grafting was used variably, including autograft, allograft, or a combination of both, and was commonly incorporated in the second stage of the Masquelet technique. Only one study utilized spacer-type constructs, in the form of custom-made 3DP antibiotic cement-coated intramedullary nails, without a primary structural regenerative role.

### Quality assessment

All 12 studies underwent quality appraisal using MINORS (Table 2), with substantial inter-reviewer agreement (k = 0.88; 95% CI 0.79–0.97). Total scores ranged from 7 to 12 out of a maximum of 16 for non-comparative studies, reflecting variability in methodological quality.

**Table 2:** MINORS score for quality of study assessment. Each item is scored out of two, with each study scored out of 16

Lead Author (Year)	Zhao (2023)	Rao (2023)	Wang (2021)	Tetsworth (2019)	Lurin (2024)	Wu (2022)	Liu (2022)	Gamieldien (2023)	Hou (2020)	Safali (2023)	Zhang (2021)	Liu (2024)
1. Clearly stated aim	2	2	0	2	2	2	2	2	2	2	2	2
2. Inclusion of consecutive patients	2	0	2	2	1	0	1	1	2	0	1	0
3. Prospective collection of data	0	0	0	0	0	0	0	0	2	0	0	0
4. Endpoints appropriate to study aim	2	2	2	2	2	2	2	2	2	2	2	2
5. Unbiased assessment of endpoints	0	0	0	0	0	0	0	0	0	0	0	0
6. Appropriate follow-up period	2	1	2	2	2	2	2	1	2	2	2	2
7. Loss to follow up < 5%	2	2	2	2	2	2	2	2	2	2	2	2
8. Prospective calculation of study size	0	0	0	0	0	0	0	0	0	0	0	0
Total MINORS Score	10	7	8	10	9	8	9	8	12	8	9	8
MINORS Score Legend	Not Reported			0	Poor Quality			1	High Quality			2

All but one study clearly stated its aims. Reporting on consecutive patient inclusion was inconsistent: four studies did not report this, four were unclear, and four explicitly confirmed inclusion of consecutive patients. All but one study were retrospective. Relevant endpoints were included across all studies; however, none specified blinded endpoint assessment. Follow-up duration was adequate (> 12 months) in ten studies, and less than 12 months in two. No patients were lost to follow-up, though prospective sample size calculation was not performed in any study.

### Upper extremity

#### Humerus

Three studies reported on 3DP custom implants for traumatic bone defects of the humerus, including a total of 7 patients [28, 30, 35]. Defect lengths were reported in six patients, with heterogeneous measurement metrics detailed further below. All defects were > 10 cm in length. Time to radiographic union was not consistently reported. Assessment metrics of bone healing were heterogeneous across studies and are detailed further below.

Humeral bone defects were due to infection and acute traumatic bone loss. Lurin et al. used polyetheretherketone (PEEK) implants in five combat casualties with defects > 10 cm. Bone bridge formation was observed on CT at 12 months, and Disability of the Arm, Shoulder and Hand (DASH) scores improved from a baseline mean of 75.3 to 49.6 (ranges not reported) at 12 months. One patient developed osteomyelitis, likely related to inadequate debridement [30]. Safali et al. reported a Ti‑6Al‑4 V implant for a single humeral defect; with restoration of elbow motion to 140 degrees of flexion and a 10-degree extension deficit, and preserved forearm rotation at three-year follow-up. Mild medial elbow pain at three years was attributed to possible chondropathy [35]. Wang et al. used an antibiotic cement‑coated titanium implant for a 12.9 cm humeral defect, achieving infection control and union within 32 weeks [28].

### Forearm

Three studies reported on traumatic bone defects of the forearm, including five patients (four radial and one ulnar) [27, 28, 35]. Defect length reporting was inconsistent. Wang et al. reported a mean defect size of 1.1 cm (range 0.6 to 1.5) in two cases [28].

Bone defects were secondary to malunion, nonunion, or infection. Rao et al. treated one distal radius malunion and one ulna mid-shaft nonunion using custom 3DP implants. Radiographic evidence of osseointegration was reported at six and three months, respectively. Postoperative motion was described as pain-free, with restoration of near-normal wrist and forearm motion [27]. Safali et al. reconstructed one radial head defect with a Ti-6Al-4 V alloy implant and reported full motion at two-year follow-up [35]. Wang et al. treated two radial defects measuring 0.6 cm and 1.5 cm with antibiotic cement-coated titanium implants, reporting infection resolution within 12 to 16 weeks and radiographic union by five to six months, without need for secondary surgery [28].

### Lower extremity

#### Femur

Seven studies reported on traumatic femoral bone defects, including 64 patients [28, 29, 31–34, 37]. Bone defects were a result of osteomyelitis, aseptic non-union, septic non-union, or acute traumatic bone loss. All studies reported on bone defect size, with a mean reported defect length of 10.7 cm (range 2.0 to 20.5).

Six studies, comprising 38 patients, reported radiographic union in all included patients at final follow-up [28, 29, 31–34]. Union definitions varied across studies and were typically based on radiographic assessment at final follow-up. Infection control was reported at latest follow-up in all patients treated for prior or active infection. Time to functional union was reported in one study, with a mean of 94.6 days (range 62 to 140) among seven patients [33].

Postoperative angular deformity was reported in two studies, with a mean of 1.8 degrees across 14 patients [29, 31]. Three studies reported a mean limb length discrepancy of 0.6 cm (range 0 to 2.7) across 19 patients [29, 31, 32]. One study reported on the preoperative and postoperative SF-36 scores and achieved significant increases (*p* ≤ 0.001) in all domains [28]. Another reported 80% of patients achieving good or excellent Harris hip and Hospital for Special Surgery knee scores, defined as scores greater than 80 [34]. One study reported a mean interval to weight bearing of 11.8 days (range 5 to 25) in five patients [32].

### Tibia

Four studies described 20 patients with traumatic tibial bone defects reconstructed using 3DP implants [28, 32, 33, 36]. Defect etiologies included osteomyelitis, aseptic nonunion, and acute trauma. Mean defect size was 10.1 cm (range 2.1 to 22.9). Radiographic union was confirmed in 13 patients. Four patients had indeterminate imaging findings at last follow-up, and three declined a planned second-stage procedure that may have influenced union outcomes. Infection control was reported at latest follow-up in all patients treated for prior or active infection.

Reported complications (*n* = 4) included superficial wound dehiscence, screw loosening, intramedullary nail breakage, and angular deformity. In one study, two patients achieved functional union at 79 and 139 days [33]. Another study documented average time to weight-bearing of 10.7 days (range 5 to 14) and limb-length discrepancy of 0.8 cm (range 0.2 to 1.2) [32]. Improvements in several SF-36 domains were reported in one series (*p* ≤ 0.001) [28].

### Calcaneus

One study reported on ten patients treated for chronic post-traumatic calcaneal osteomyelitis using titanium 3DP implants (mean defect volume 18.0 cm³; range 3 to 24) [26].

A two-stage reconstruction was performed consisting of initial debridement with PMMA spacer and flap coverage, followed by titanium cage reconstruction with rib autografting. All patients achieved radiographic union (mean 4.3 months; range 3 to 8). Two infections occurred and resolved after repeat debridement and polymethyl methacrylate (PMMA) replacement.

Functional outcomes were excellent, with mean Maryland Foot Score 92 (range 86 to 98) and American Orthopedic Foot and Ankle Society (AOFAS) hindfoot score 89.8 (range 83 to 100) at 12 months.

**Fig. 3:**
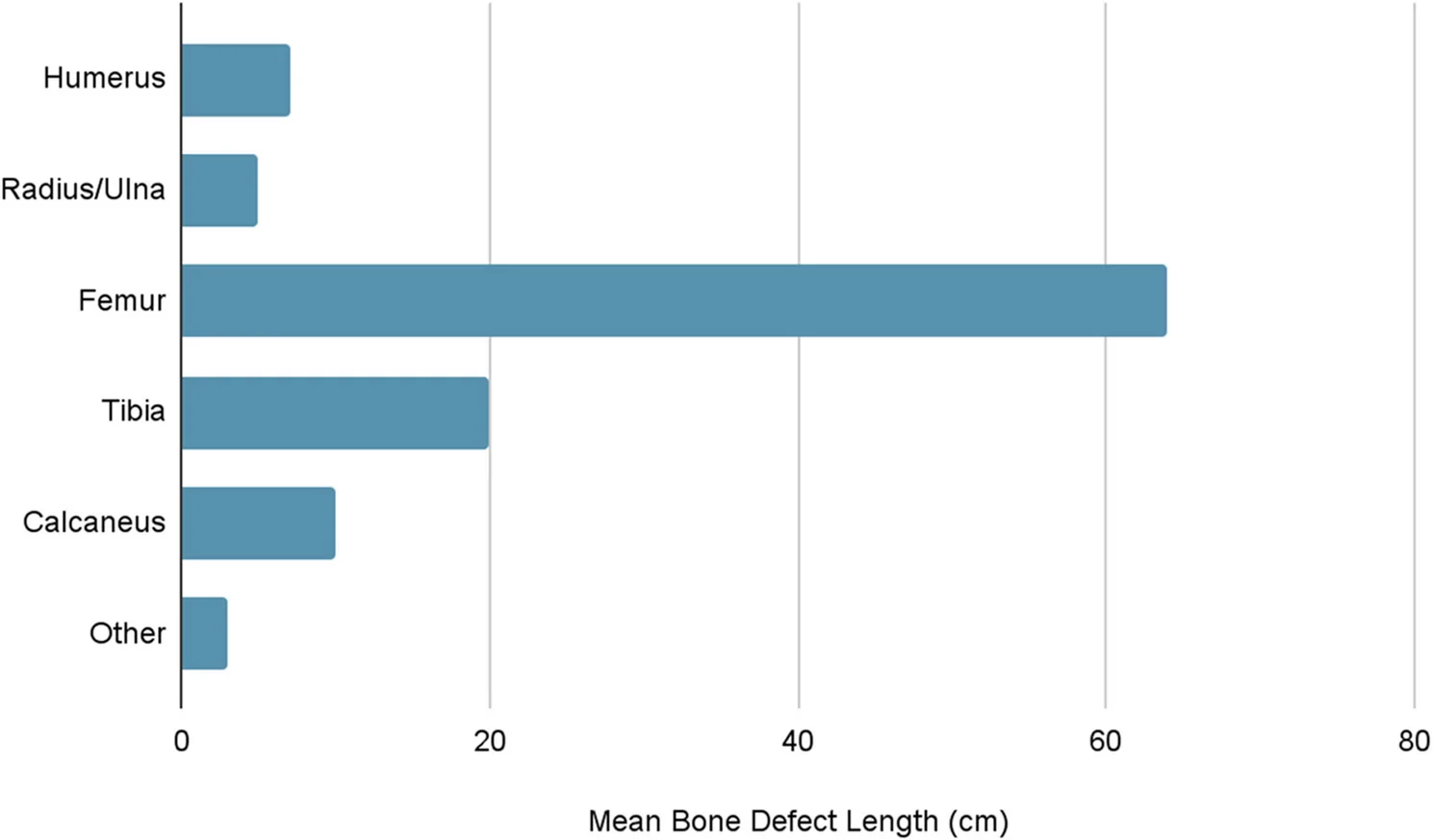
Mean size of bone defects by anatomic location. *Humerus data only reports “>10 cm”. **Ulna/radius defect size was only reported on two out of five patients. ***Calcaneus defect size provided in cm3

A summary of clinical outcomes by anatomical site is displayed in Table 3. Figure 3 illustrates the mean size of critical bone defects by anatomic location.

**Table 3:** Summary of clinical outcomes by anatomical site

Anatomical Site	No. of Patients	Radiographic Union Achieved (%)	Complications	Functional Outcomes
Femur [28, 29, 31–34, 37]	64	94%	Angular deformity, minor hardware issues	Improved SF-36, Harris Hip Score, HSS Knee Score, early weight-bearing
Tibia [28, 32, 33, 36]	20	65% confirmed, 20% indeterminate	Wound dehiscence, screw loosening, nail breakage, angular deformity	Improved SF-36, early weight-bearing
Calcaneus [26]	10	100%	2 infections treated successfully	High Maryland Foot Score and AOFAS Hindfoot Score
Humerus [28, 30, 35]	7	Bone bridge formation in PEEK, union in Ti cases	1 osteomyelitis (PEEK), elbow pain (Ti)	Improved DASH scores, good range of motion
Forearm [27, 28, 35]	5	100%	None reported	Full or near-full range of motion

*Ti* titanium, *PEEK* polyetheretherketone, *AOFAS* American Orthopedic Foot and Ankle Society, *SF-36* 36-Item Short Form Survey, *DASH* Disabilities of the Arm, Shoulder, and Hand

### Materials and manufacturing

This review included nine studies reporting the use of titanium or titanium alloy implants in 92 patients [26, 28, 29, 32–37]. These implants predominantly functioned as osseointegrative structural scaffolds for intercalary defect reconstruction. Several studies incorporated adjunctive bone grafting, including autograft, allograft, or combined grafting strategies, particularly in the context of staged Masquelet reconstruction. All studies reported satisfactory bone growth and healing, with no implant failures. Wu et al. described the use of tantalum scaffolds without additional bone grafting in nine patients and reported radiographic evidence of osseointegration without implant-related complications [31]. Lurin et al. utilized PEEK lattice scaffolds in conjunction with allograft bone in five patients with humeral defects greater than 10 cm, reporting bone bridge formation at 12 months and improvement in DASH scores (mean improvement from 75.3 to 49.6). One patient developed osteomyelitis [30].

Few studies provided detailed reporting on manufacturing methods. Three studies [33, 34, 37] reported the use of EBM (brand not mentioned) and two studies [32, 36] reported the use of SLM (Arcam EBM, Gothenburg, Sweden).

### Complications

For the purposes of this review, catastrophic implant failure was defined as implant fracture, structural collapse, or mechanical loosening requiring implant removal or revision.

Reported complications included two infections managed with debridement [26]; one case of protective plate failure with maintained intramedullary nail stability [28]; one locking screw loosening and one angulation deformity [32]; one intramedullary nail breakage, two broken interlocking screws, and one loose interlocking screw [36]; one case of limited knee range of motion (ROM), one superficial stitch abscess, and one locking screw loosening revised without sequelae [29]; and one case of chondropathy and degenerative changes attributed to possible implant-cartilage incompatibility [35].

No implant fractures, structural collapses, or mechanical failures requiring implant removal were reported in the included studies.

## Discussion

Across the 12 studies included in this review, encompassing 108 patients with trauma-related bone defects, custom 3DP implants were associated with frequently reported radiographic union and generally favorable functional outcomes across multiple anatomical sites. Titanium and titanium alloy implants were the most commonly utilized materials and were consistently described as demonstrating satisfactory osseointegration and structural performance. While tantalum implants also showed encouraging results, their reported use remains limited. Reported complication rates were generally manageable, and no cases of catastrophic implant failure were described. However, interpretation of these findings is limited by the predominance of small retrospective case series and variable follow-up duration.

### Clinical insights by anatomical site

Lower extremity reconstructions, particularly of the femur and tibia, were frequently reported to achieve structural restoration with early weight bearing and acceptable functional recovery. In the calcaneus, where complex anatomy presents reconstructive challenges, custom implants were described with favorable short-term functional scores. Upper extremity applications were more heterogeneous. PEEK implants in humeral defects demonstrated bone bridging but were associated with infection in one case, whereas titanium-based implants in forearm applications were reported to achieve radiographic integration and restoration of motion.

### Outcome comparison with conventional techniques

The outcomes summarized in this review suggest that custom 3DP implants may represent a feasible reconstructive option for selected traumatic bone defects. Across anatomical sites, radiographic union was frequently reported; however, union definitions, follow-up duration, and adjunctive procedures varied substantially between studies. Functional improvements were described in several series, although outcome measures were not standardized.

Compared with established reconstructive strategies such as the Masquelet technique, distraction osteogenesis, or vascularized autografts, 3DP implants offer the theoretical advantages of anatomical customization and structural stability [7, 16]. However, direct comparison is not currently possible, as no included studies were comparative in design. Reported complication rates appear comparable to those described in existing literature for conventional techniques, but such comparisons must be interpreted cautiously given the absence of controlled data; therefore, the relative effectiveness of 3DP implants compared with Masquelet reconstruction, distraction osteogenesis, vascularized autografting, or structural allografting remains undefined [7, 38, 39].

An important finding of this review is the heterogeneity in how 3DP constructs were utilized across studies. Most implants functioned as osseointegrative structural scaffolds for intercalary defect reconstruction, whereas spacer-type constructs were infrequently reported and were primarily used for temporary defect management rather than definitive reconstruction. Many reconstructions also incorporated adjunctive autograft, allograft, staged Masquelet reconstruction, or other biological augmentation, limiting attribution of union, infection control, and functional recovery to the 3DP implant alone. These distinctions highlight that 3DP implants are not a uniform intervention, but rather represent a spectrum of reconstructive strategies with differing biological and mechanical roles, which complicates direct comparison between studies and makes subgroup comparison by graft use unreliable.

### Materials and manufacturing

Most research on 3DP orthopaedic implants has focused on biomechanical performance, bioactivity, and osseointegration in preclinical models. Titanium alloys (Ti-6Al-4 V) and commercially pure titanium, which are widely used in conventional orthopaedic implants, remain the most commonly reported materials because of their biocompatibility, mechanical strength, and corrosion resistance [15].

Additive manufacturing enables the creation of porous microstructures that promote bone ingrowth and implant fixation [15, 40]. A recognized limitation of titanium-based implants is the potential for stress shielding due to differences between implant and bone modulus of elasticity [16]. Beta-type titanium alloys have been proposed as a potential strategy to improve mechanical compatibility [16].

Tantalum is another material used in orthopaedic implants and demonstrates biocompatibility and corrosion resistance with mechanical properties similar to cancellous bone [16]. Preclinical studies have suggested enhanced osteoinductive potential and bone integration compared with titanium [41–43]. However, its broader use remains limited by manufacturing complexity and cost [16].

PEEK has been widely used in maxillofacial and spinal applications. Although radiolucent and mechanically favorable, it demonstrates reduced bioactivity and osseointegration compared with titanium-based materials in preclinical studies [17, 44]. In this review, osteomyelitis was reported in one of five humeral cases treated with polyetheretherketone implants; however, given the small sample size, no definitive conclusions can be drawn regarding material-specific infection risk.

Two primary manufacturing methods were reported. EBM produces high-strength implants with low impurity levels but may yield rougher surface finishes requiring post-processing [15, 16]. SLM offers high three-dimensional accuracy and favorable mechanical properties, although it may be associated with longer production times and higher cost [15, 16]. Based on the limited number of studies reporting manufacturing techniques, there is insufficient evidence to determine whether manufacturing method influences clinical outcomes.

### Barriers to clinical adoption

Several barriers may limit broader clinical adoption of 3DP implants for trauma-related bone defects. These include production costs, regulatory requirements for patient-specific devices, surgeon familiarity with design workflows, and manufacturing timelines that may limit applicability in acute trauma settings [16, 17]. None of the included studies reported detailed implant production costs, reimbursement data, or formal cost-effectiveness analyses, and the economic value of these implants remains undefined. In addition, heterogeneity in outcome reporting, limited long-term follow-up, uncertain revision implications, and likely concentration of this technology in specialized centers reduce the ability to establish standardized performance benchmarks or draw conclusions about broader implementation and access.

### Limitations

This review is limited by the small number of available studies and the predominance of Level IV evidence, as most included reports were retrospective non-comparative case series [45]. The absence of prospective or randomized comparative trials limits causal inference regarding effectiveness relative to established reconstructive techniques. Follow-up duration varied widely, and long-term durability beyond five years remains largely undefined. Although most studies provided at least 12 months of follow-up by MINORS criteria, follow-up was shorter or incompletely reported in a minority of studies and remains insufficient to assess long-term implant durability. The available evidence therefore supports only short- to mid-term assessment of union, early functional recovery, and complications, while late infection, stress shielding, implant loosening, revision risk, and durability over decades remain undefined.

Substantial heterogeneity in defect characteristics, adjunctive procedures, union definitions, and outcome reporting limited quantitative synthesis. Union and healing outcomes were also assessed heterogeneously across studies, with variable use of radiographs, computed tomography, clinical criteria, functional recovery, and descriptive findings such as implant incorporation or new bone formation. This variability in terminology, imaging modality, clinical criteria, and follow-up timing limited direct comparison of union rates across studies. Important baseline patient and injury characteristics relevant to bone healing, including comorbidities, smoking status, infection burden, timing of reconstruction, and soft-tissue status, were inconsistently reported across studies. This limited assessment of patient-level confounding across reconstructive strategies. Potential publication bias and early adopter bias must also be considered, as reported outcomes may preferentially reflect specialized centers with favorable early experience. Finally, cost data and standardized outcome reporting, including consistent functional outcome measures, complication definitions, and follow-up time points, were largely absent, limiting assessment of cost-effectiveness, functional recovery, safety comparisons, and generalizability.

### Research gaps and future directions

Future research should prioritize well-designed prospective studies and randomized controlled trials comparing 3DP implants to conventional techniques. The development of consensus on reporting standards and outcome metrics will enhance comparability across studies. Advances in materials science, such as bioactive coatings and biodegradable scaffolds, may further improve biological integration and clinical outcomes [46, 47]. These approaches are relevant to the broader field of bone-defect reconstruction, but many currently reported biodegradable scaffold studies are preclinical [46, 47]. Finally, efforts to streamline regulatory pathways and reduce costs will be essential to facilitate broader adoption of this promising technology.

## Conclusions

Custom 3DP implants represent an emerging reconstructive option for selected traumatic bone defects. Available evidence, derived primarily from small retrospective case series, suggests feasibility with reported radiographic union and functional improvement across several anatomical sites. However, certainty of evidence remains low. Larger prospective comparative studies with standardized outcome reporting are required to clarify long-term durability, cost-effectiveness, and appropriate indications relative to established reconstructive techniques.

## Supplementary Information

Supplementary Material 1: Appendix 1. Table A1. Medline search queries. Table A2. Embase search queries. Table A3. Scopus search queries. Table A4. Preferred Reporting Items for Systematic reviews and Meta-Analyses extension for Scoping Reviews (PRISMA-ScR) Checklist.

## Data Availability

All data analyzed during this study were derived from previously published articles and are available in the public domain.
