# Supplementary material for: Clinical outcomes and surgical applications of 3D-printed patient-specific implants for traumatic bone defects: a scoping review
Source: J Orthop Surg Res. 2026 Jul 29;21:604. doi: 10.1186/s13018-026-07064-z (PMC13700128; doi:10.1186/s13018-026-07064-z)
Supplement: Supplementary file 1 [file 13018_2026_7064_MOESM1_ESM.docx]

**Appendix A**

Table A1. Medline search queries

| **#** | **Search Query** | **Results** |
| --- | --- | --- |
| 1 | bone regeneration.mp. or Bone Regeneration/ | 29183 |
| 2 | Osteogenesis, Distraction/ or osteogenesis.mp. or Osteogenesis/ | 64617 |
| 3 | tissue scaffold.mp. or Tissue Scaffolds/ | 30322 |
| 4 | bone defect.mp. | 9264 |
| 5 | Bone Transplantation/ or bone transplant.mp. | 33438 |
| 6 | 4 and 5 | 1052 |
| 7 | bone.mp. or "Bone and Bones"/ | 1094454 |
| 8 | tissue engineering.mp. or Tissue Engineering/ | 76080 |
| 9 | 8 and 9 | 22960 |
| 10 | Printing, Three-Dimensional/ or 3d print.mp. | 13177 |
| 11 | Three-Dimensional Print*.mp. | 4296 |
| 12 | Bioprinting/ or bioprint*.mp. | 5186 |
| 13 | 1 or 2 or 3 or 4 or 5 or 6 or 9 | 146317 |
| 14 | 10 or 11 or 12 | 18725 |
| 15 | 13 and 14 | 4112 |
| 16 | limit 15 to humans | 1993 |

Table A2. Embase search queries

| **#** | **Search Query** | **Results** |
| --- | --- | --- |
| 1 | bone regeneration.mp. or bone regeneration/ | 38956 |
| 2 | osteogenesis.mp. or bone development/ | 73751 |
| 3 | tissue scaffold.mp. or tissue scaffold/ | 23616 |
| 4 | bone defect.mp. or bone defect/ | 22841 |
| 5 | bone transplantation/ or bone graft/ or bone transplant*.mp. | 51105 |
| 6 | bone graft.mp. | 44003 |
| 7 | bone tissue/ or engineered bone graft/ or bone growth/ or engineered bone allograft/ or bone prosthesis/ or synthetic bone graft/ | 45191 |
| 8 | three dimensional printing/ or three-dimensional print*.mp. | 27506 |
| 9 | 3D print*.mp. | 28129 |
| 10 | bioprinting/ or bioprint*.mp. | 7470 |
| 11 | tissue engineering/ and bone tissue/ | 5466 |
| 12 | 1 or 2 or 3 or 4 or 5 or 6 or 7 | 198895 |
| 13 | 8 or 9 or 10 | 40924 |
| 14 | 12 and 13 | 5673 |
| 15 | limit 14 to human | 3427 |

Table A3. Scopus search queries

**Scopus – 2816 articles**

( TITLE-ABS-KEY ( bone AND regeneration ) OR TITLE-ABS-KEY ( osteogenesis ) OR TITLE-ABS-KEY ( bone AND transplantation ) OR TITLE-ABS-KEY ( bone AND defect ) OR TITLE-ABS-KEY ( bone AND tissue AND engineering ) AND TITLE-ABS-KEY ( three-dimensional AND printing ) OR TITLE-ABS-KEY ( 3d AND printing ) OR TITLE-ABS-KEY ( bioprinting ) ) AND ( LIMIT-TO ( EXACTKEYWORD , "Human" ) )

Table A4. Preferred Reporting Items for Systematic reviews and Meta-Analyses extension for Scoping Reviews (PRISMA-ScR) Checklist

| **Section** | **Item** | **PRISMA-ScR checklist item** | **Reported on page/location** |
| --- | --- | --- | --- |
| Title | 1. Title | Identify the report as a scoping review. | p. 1, title |
| Abstract | 2. Structured summary | Provide a structured summary including, as applicable, background, objectives, eligibility criteria, sources of evidence, charting methods, results, and conclusions. | pp. 2-3, Abstract |
| Introduction | 3. Rationale | Describe the rationale for the review in the context of what is already known and explain why a scoping review approach is appropriate. | pp. 4-5, Introduction |
| Introduction | 4. Objectives | Provide an explicit statement of the questions and objectives being addressed with reference to key elements. | p. 5, Introduction |
| Methods | 5. Protocol and registration | Indicate whether a review protocol exists; state where it can be accessed; and provide registration information if available. | p. 6, Methods |
| Methods | 6. Eligibility criteria | Specify characteristics of sources of evidence used as eligibility criteria and provide a rationale. | p. 6, Eligibility Criteria |
| Methods | 7. Information sources | Describe all information sources in the search and the date the most recent search was executed. | p. 6, Search Strategy; Appendix A pp. 27-28 |
| Methods | 8. Search | Present the full electronic search strategy for at least one database, including limits used, such that it could be repeated. | p. 6; Appendix A pp. 27-28 |
| Methods | 9. Selection of sources of evidence | State the process for selecting sources of evidence included in the scoping review. | p. 6, Study Selection; p. 8 and Figure 1 p. 23 |
| Methods | 10. Data charting process | Describe the data charting methods and whether data charting was done independently or in duplicate. | pp. 6-7, Data Extraction |
| Methods | 11. Data items | List and define all variables for which data were sought and any assumptions or simplifications. | pp. 6-7, Data Extraction |
| Methods | 12. Critical appraisal of individual sources of evidence | If done, provide a rationale, methods, and how appraisal information was used in synthesis. | p. 7, Quality Assessment; Table 2 p. 26 |
| Methods | 13. Synthesis of results | Describe the methods of handling and summarizing the charted data. | p. 7, Data Synthesis and Statistical Analysis |
| Results | 14. Selection of sources of evidence | Give numbers screened, assessed for eligibility, and included, with reasons for exclusions at each stage, ideally using a flow diagram. | p. 8, Study Selection; Figure 1 p. 23 |
| Results | 15. Characteristics of sources of evidence | For each source of evidence, present characteristics for which data were charted and provide citations. | p. 8, Study Characteristics; Table 1 pp. 24-25 |
| Results | 16. Critical appraisal within sources of evidence | If done, present data on critical appraisal of included sources of evidence. | pp. 8-9, Quality Assessment; Table 2 p. 26 |
| Results | 17. Results of individual sources of evidence | For each included source of evidence, present relevant charted data related to review questions and objectives. | pp. 9-12, anatomical results; Table 1 pp. 24-25; Table 3 pp. 26-27 |
| Results | 18. Synthesis of results | Summarize and/or present charting results as they relate to the review questions and objectives. | pp. 8-12, Results; Table 3 pp. 26-27; Figures 2-3 pp. 23-24 |
| Discussion | 19. Summary of evidence | Summarize the main results, link to review questions/objectives, and consider relevance to key groups. | pp. 13-15, Discussion |
| Discussion | 20. Limitations | Discuss the limitations of the scoping review process. | pp. 15-16, Limitations |
| Discussion | 21. Conclusions | Provide a general interpretation of results with respect to the review questions/objectives and potential implications or next steps. | pp. 16-17, Research Gaps and Future Directions; Conclusions |
| Funding | 22. Funding | Describe sources of funding for included sources of evidence and sources of funding for the scoping review; describe funder roles. | p. 7, Data Availability and Ethics Statement; p. 18, Declarations |
